# PFuji-Size dataset: A collection of images and photogrammetry-derived 3D point clouds with ground truth annotations for Fuji apple detection and size estimation in field conditions

**DOI:** 10.1016/j.dib.2021.107629

**Published:** 2021-11-24

**Authors:** Jordi Gené-Mola, Ricardo Sanz-Cortiella, Joan R. Rosell-Polo, Alexandre Escolà, Eduard Gregorio

**Affiliations:** Research Group in AgroICT & Precision Agriculture - GRAP^1^, Department of Agricultural and Forest Engineering, Universitat de Lleida (UdL) – Agrotecnio-CERCA Center, Lleida, Catalonia, Spain

**Keywords:** Fruit detection, Fruit size, 3D object detection, Structure-from-motion, Multi-view stereo, Agricultural robotics, Precision agriculture

## Abstract

The PFuji-Size dataset is comprised of a collection of 3D point clouds of Fuji apple trees (*Malus domestica* Borkh. cv. Fuji) scanned at different maturity stages and annotated for fruit detection and size estimation. Structure-from-motion and multi-view stereo techniques were used to generate the 3D point clouds of 6 complete Fuji apple trees containing a total of 615 apples. The resulting point clouds were 3D segmented by identifying the 3D points corresponding to each apple (3D instance segmentation), obtaining a single point cloud for each apple. All segmented apples were labelled with ground truth diameter annotations. Since the data was acquired in field conditions and at different maturity stages, the set includes different fruit diameters -from 26.9 mm to 94.8 mm- and different fruit occlusion percentages due to foliage. In addition, 25 apples were photographed 360° in laboratory conditions, obtaining high resolution 3D point clouds of this sub-set. To the best of the authors’ knowledge, this is the first publicly available dataset for apple size estimation in field conditions. This dataset was used to evaluate different fruit size estimation methods in the research article titled “In-field apple size estimation using photogrammetry-derived 3D point clouds: comparison of 4 different methods considering fruit occlusion” (Gené-Mola et al., 2021).

## Specifications Table

**Table:** 

Subject	Agriculture Engineering, Computer Vision and Pattern Recognition
Specific subject area	Precision Agriculture, Fruit Detection, Fruit Size Estimation, Machine Learning, Agricultural Robotics
Type of data	Images 3D coloured point clouds 3D instance (fruit) segmentation annotations Fruit size (diameter) annotations
How data were acquired	- Ground truth fruit diameters were measured using a Vernier calliper. - Raw images were taken using an EOS 60 DSLR Camera (Canon Inc. Tokyo, Japan) with an 18 MP CMOS APS-C sensor and an EFS 24 mm f/2.8 STM lens (38 mm film-equivalent focal length). - 3D point clouds were generated with Agisoft Professional Metashape software (v1.6.4, Agisoft LLC, St. Petersburg, Russia). - 3D apple segmentation ground truth was manually annotated by identifying the 3D points corresponding to each apple with CloudCompare software (Cloud Compare [GPL software] v2.9 Omnia).
Data format	Raw images: *JPG* Apple tree point clouds: *LAZ* Apple segmented point clouds: *TXT* Apple size and location annotations: *TXT*
Parameters for data collection	*- Image acquisition*: Images were acquired from a distance of 2.5 m with respect to the tree centres with a horizontal and vertical overlap between consecutive images higher than 75% and 85%, respectively. *- 3D point cloud reconstruction*: The parameters set to the Agisoft Professional Metashape Software were: camera alignment accuracy, “highest”; key point limit, “40000”; tie point limit, “4000”; dense cloud quality, “high”; depth filtering, “Mild”.
Description of data collection	The diameter of 615 Fuji apples was manually measured with the calliper. The 6 trees containing the measured apples were then photographed from different positions in order to reconstruct the corresponding 3D model using structure-from-motion and multi-view stereo techniques. The resulting 3D point clouds were manually segmented by identifying the points of all measured apples in the clouds (3D instance segmentation masks). Each segmented apple was pair-wised with its corresponding ground truth diameter. In addition, a set of 25 additional Fuji apples were picked and scanned in laboratory conditions.
Data source location	City/Town/Region: Agramunt, Catalonia Country: Spain GNSS coordinates for collected samples/data: E = 336297 m, N:= 4623494 m, 312 m a.s.l., UTM 31T - ETRS89
Data accessibility	Repository name: Dataverse Data identification number: https://doi.org/10.34810/data141 DOI: https://doi.org/10.34810/data141 Direct URL to data: https://dataverse.csuc.cat/dataset.xhtml?persistentId=doi:10.34810/data141
Related research article	J. Gené-Mola, R. Sanz-Cortiella, J.R. Rosell-Polo, A. Escolà, E. Gregorio. 2021. In-field apple size estimation using photogrammetry-derived 3D point clouds: comparison of 4 different methods considering fruit occlusions., Comput. Electron. Agric. 188 (2021) 106343. https://doi.org/10.1016/j.compag.2021.106343[1]

## Value of the Data

• First publicly available dataset for fruit detection and size estimation containing photogrammetry-derived 3D point clouds annotated with apple segmentation masks and fruit diameter ground truth. This dataset differs from previously published datasets [2], [3], [4], [5] in: (1) data acquired at different maturity stages; (2) fruit location annotations in the form of 3D instance (apple) segmented point clouds; (3) providing the actual diameter of all apples labelled in the point cloud.
• The precision agriculture community can benefit from these data to test new fruit detection and size estimation methodologies, with applications in yield prediction [6,7] and automated harvesting [8,9].
• New fruit size estimation methods can be tested either using the original point clouds -containing a complete representation of 6 apple trees- or using the provided ground truth apple segmentation masks (640 apple point clouds).
• The dataset includes 615 apples scanned under in-field conditions at two different phenology stages: green apples at 70% of final size and red apples at advanced ripening. The data includes different fruit diameters -from 26.9 mm to 94.8 mm- and different fruit occlusion percentages -from 1.4 % to 99 %-.
• An additional set of 25 apples with diameters from 39 mm to 97 mm were scanned in laboratory conditions. The resulting high resolution 3D point clouds (provided in the dataset) are a 360° complete representation of the apples.
• This dataset allows all methods and results reported in the corresponding research paper [1] to be reproduced.

## 1 Data Description

This dataset includes three different data sub-sets. The first, called *Orch2018,* includes 303 apples captured under in-field conditions on October 3rd, 2018, when apples were at advanced ripening growth stage (reddish coloured). The second, called *Orch2020*, includes 312 apples captured under in-field conditions on July 16th, 2020, when apples were about 70% their final size (green coloured). Finally, the third sub-set of data, called *Lab2020*, was acquired on October 2nd, 2020. In this case, 25 apples of different sizes were imaged in laboratory conditions.

**Fig. 1:**
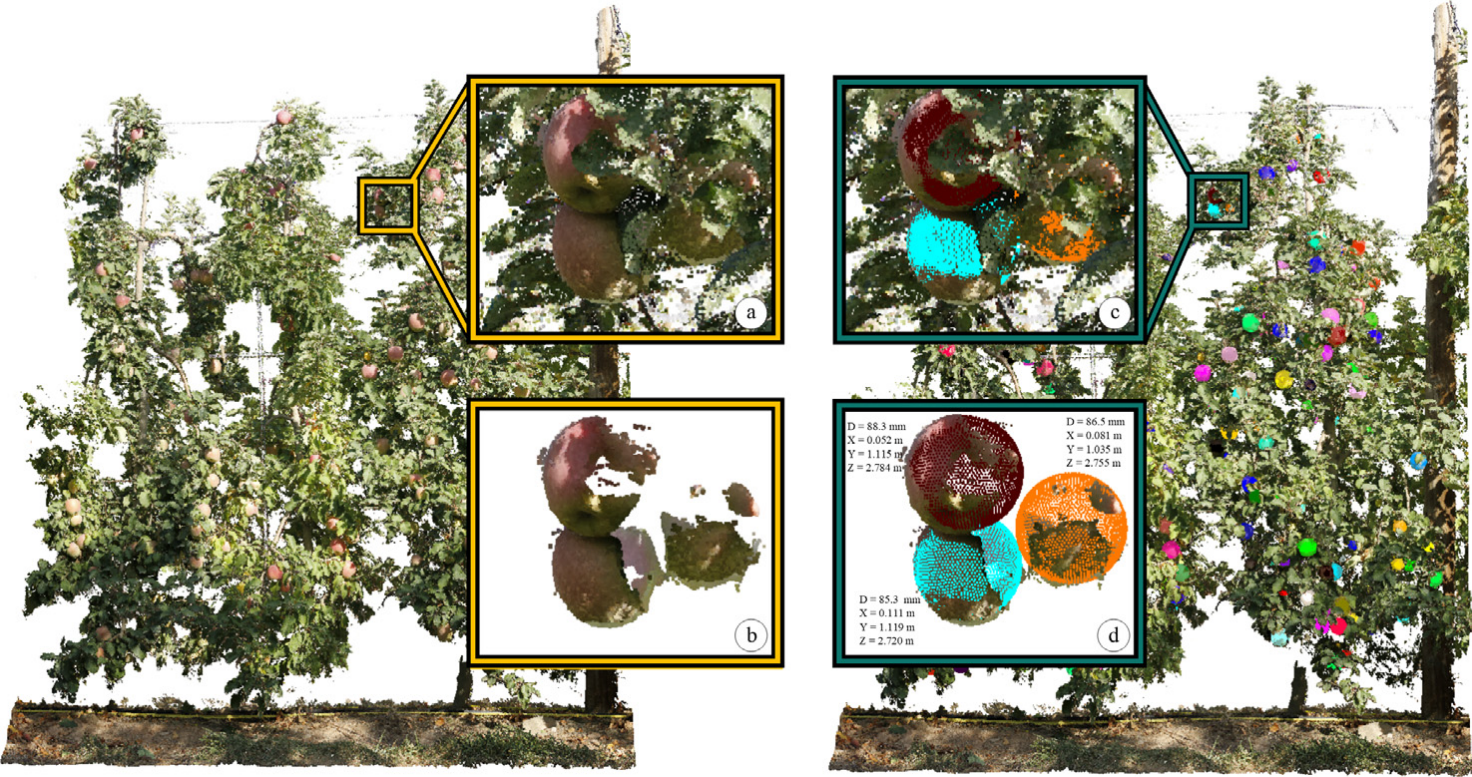
Point clouds and annotations provided in the Orch2018 set. Point cloud of the 3 captured trees (background left), zoom-in on 3 apples (a) and the corresponding apple segmented points (b). The same point cloud with apple locations and diameter ground truth (background right) and zoom-in on 3 apples (c). For each apple point cloud the apple centre [X,Y,Z] and diameter ground truth are provided (d). These ground truth annotations are illustrated in (c) and (d) through the red, orange and blue spheres.

The data provided in this dataset include: (1) raw images used to generate the 3D point clouds with structure-from-motion and multi-view stereo techniques; (2) the 3D point clouds of the captured scene -3 apple trees in *Orch2018* and 3 apple trees in *Orch2020*- (Fig. 1a); (3) the segmented point clouds for all apples present in the captured trees (Fig. 1b); (4) apple centroid ([X, Y, Z] coordinate) and diameter ground truth (Fig. 1c and d).

This dataset differs from previously published datasets [3,4] in:

• Data acquired in a different set of trees, different dates and at different phenology stages. While the data published in [3,4] included 11 trees scanned in 2017 when fruits were ripe, the present dataset was acquired in a different set of trees, at two different dates (2018 and 2020) and different maturity stages: green apples at 70% of their final size (*Orch2020*) and red apples at advanced ripening (*Orch2018*).
• Different type of fruit detection and location annotations. Previous published datasets included 2D instance segmented images [4], and/or 3D location annotations in the form of 3D bounding boxes [3,4]. Alternatively, the present dataset provides the 3D fruit location annotations in the form of 3D instance (apple) segmented point clouds. This 3D instance segmentation ground truth could not be reproducible from the annotations provided in [4] because the segmentation masks provided in the present dataset refer to the 3D point cloud, while the segmentation masks provided in [4] referred to 2D images.
• The present dataset provides the ground truth diameter and the apple centre [X,Y,Z] of all fruits in the point clouds for fruit size estimation.

**Fig. 2:**
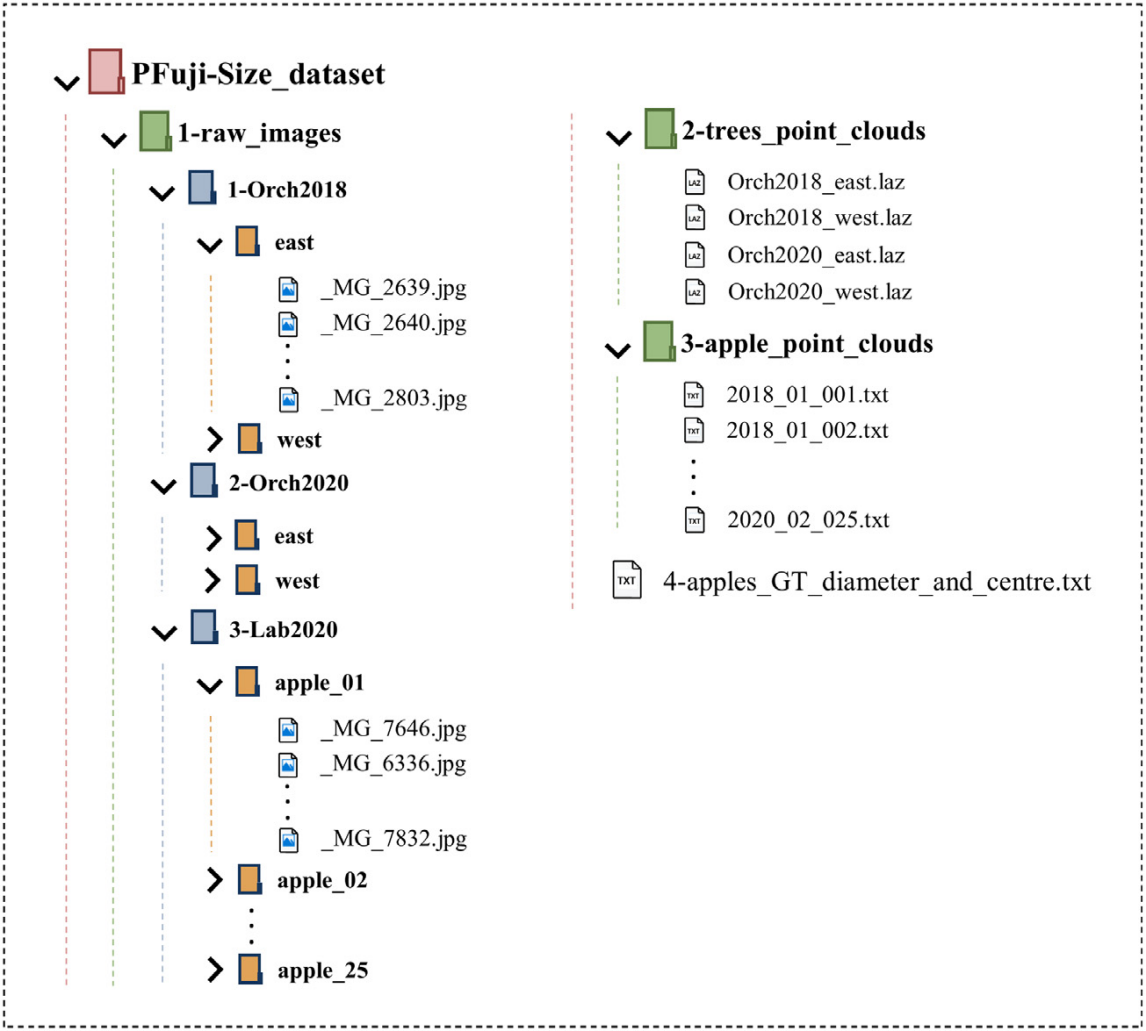
Dataset files and folders structure.

The dataset can be downloaded from https://doi.org/10.34810/data141. Once unpacked, the data is structured as shown in Fig. 2. The first folder, */PFuji-Size_dataset/1-raw_images* (27.2 GB), includes all raw images used to generate the point clouds. Images were saved in *.JPG* format and have a resolution of 5184 × 3456 pixels. A total of 168 and 180 images were acquired respectively from the eastern and western sides of the tree row captured in *Orch2018*, and a total of 228 and 280 images respectively from the eastern and western sides of the tree row captured in *Orch2020*. The images acquired in laboratory conditions are placed in the */PFuji-Size_dataset/1-raw_images/3-Lab2020* folder, which includes a total of 180 images per apple (25 apples * 180 images/apple = 4500 images). Section 2 describes in greater detail the methodology followed to acquire these images.

The second folder, */PFuji-Size_dataset/2-trees_point_clouds* (400 MB) (Fig. 2), includes the point clouds of trees captured under in-field conditions. For each set of data -*Orch2018* and *Orch2020*- two point cloud files were generated: one obtained from the eastern side of the tree row and the other from the western side. Each of these point clouds was saved in *.LAZ* format and contains between 8.8 M and 9.6 M points to 3D represent the three trees in each data sub-set: three trees in *Orch2018* and three trees in *Orch2020*. The *LAZ* format is the compressed version of the standard *LAS* file format used for point cloud data records [10]. *LAZ* files can be directly opened by using a point cloud editing software such as CloudCompare (Cloud Compare [GPL software] v2.9 Omnia), or can be decompressed to *LAS* format by using the LASzip software https://laszip.org/.

Each pair of east-west point clouds were merged and segmented. Then, points corresponding to each apple were aggregated and saved, obtaining a single point cloud per apple (3D apple segmentation masks). The resulting apple point clouds (315 apples) were saved in the third folder, named */PFuji-Size_dataset/3-apple_point_clouds* (229 MB) (Fig. 2). In addition, this folder also contains the 25 apple point clouds generated in laboratory conditions (*Lab2020*). All these apple point clouds were saved in *.TXT* files, where each row corresponds to a single 3D point, giving the information of [x, y, z, R, G, B], where, [x, y, z] is the point position in global coordinates, and [R,G,B] is the point colour with 8-bit resolution values ranging from 0 to 255.

**Fig. 3:**
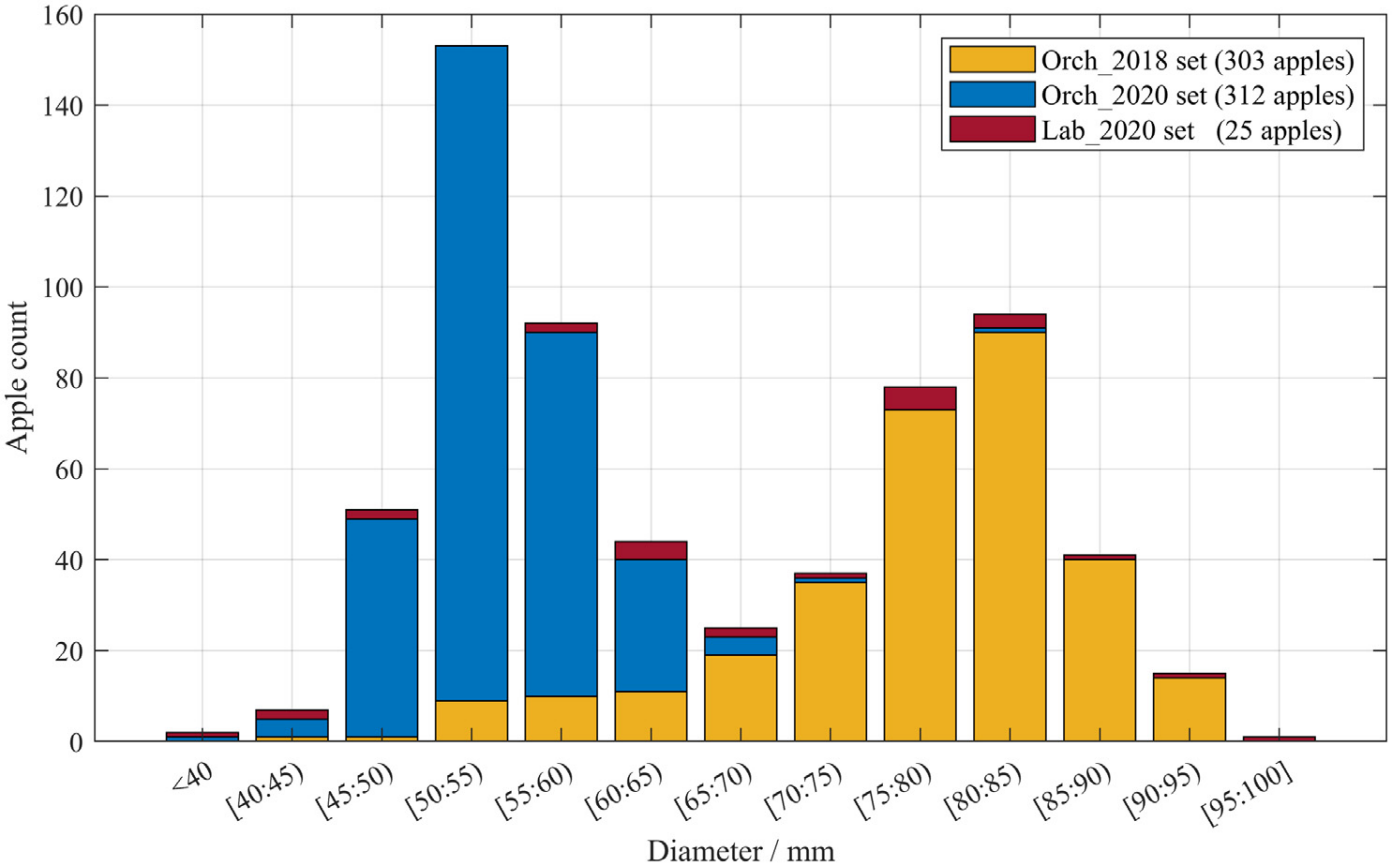
Apple ground truth diameter histogram. The number of apples at different diameter intervals for Orch2018, Orch2020 and Lab2020 sub-sets are represented in orange, blue and red, respectively.

Finally, the file */PFuji-Size_dataset/4-apples_GT_diameter_and_centre.txt* (46 kB) includes the ground truth fruit diameters and location. Each row corresponds to an apple annotation giving the following information: apple identification name (Apple_ID), diameter [mm], apple centre X coordinate [m], apple centre Y coordinate [m], and apple centre Z coordinate [m]. Fig. 3 illustrates a histogram of the distribution of the ground truth diameters measured in each data sub-set. Apples measured in *Orch2018* (303 apples) had a mean diameter of 77.4 mm and a standard deviation of 9.4 mm. Apples measured in *Orch2020* (312 apples) had a mean diameter of 54.1 mm and a standard deviation of 5.1 mm. Finally, the distribution of apples scanned in *Lab2020* (25 apples) shows a similar number of samples in each diameter interval, including apple diameters between 39 and 97 mm.

## 2 Experimental Design, Materials and Methods

The PFuji-Size dataset was acquired in a Fuji apple (*Malus domestica* Borkh. cv. Fuji) orchard located in Agramunt, Catalonia, Spain (latitude: 41° 44′ 47.07′′ N; longitude: 1° 01′ 52.23′′ E; 312 m a.s.l. ETRS89). The captured trees were trained in a tall spindle system and had a maximum canopy height of 3.5 m and width of 1.5 m, approximately.

**Fig. 4:**
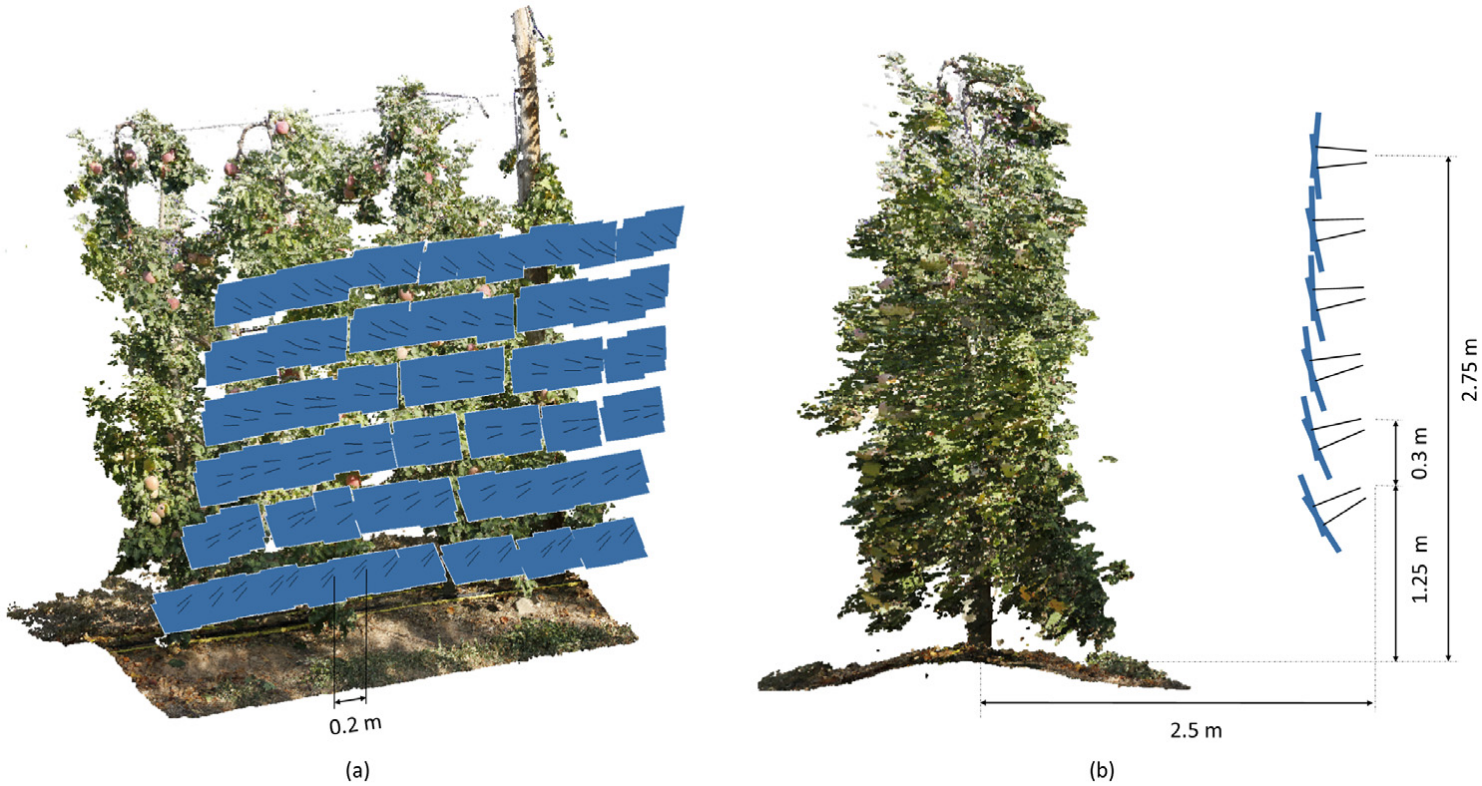
Photographic process layout followed to capture trees from Orch2018 and Orch2020. (a) Isometric view of Orch2018 scene showing the horizontal separation between neighbouring images. (b) Transversal view of trees scanned in Orch2018 showing the camera positions: height and distance from the centre of the tree row.

The camera used was an EOS 60 DSLR with an 18 MP CMOS APS-C sensor and an EFS 24 mm f/2.8 STM lens (Canon Inc. Tokyo, Japan). A total of 348 and 508 images were taken to capture the trees in the *Orch2018* and *Orch2020* sets, respectively. Images were acquired following the experimental layout illustrated in Fig. 4. The distance between two consecutive horizontal camera positions was 0.2 m (Fig. 4a). From each horizontal position, a total of 12 photographs were acquired from 6 different heights (two photographs from each height) (Fig. 4b). The distance between two consecutive vertical camera positions was 0.3 m (Fig. 4b). With this configuration, the overlap between horizontal and vertical consecutive images was higher than 85% and 75%, respectively.

**Fig. 5:**
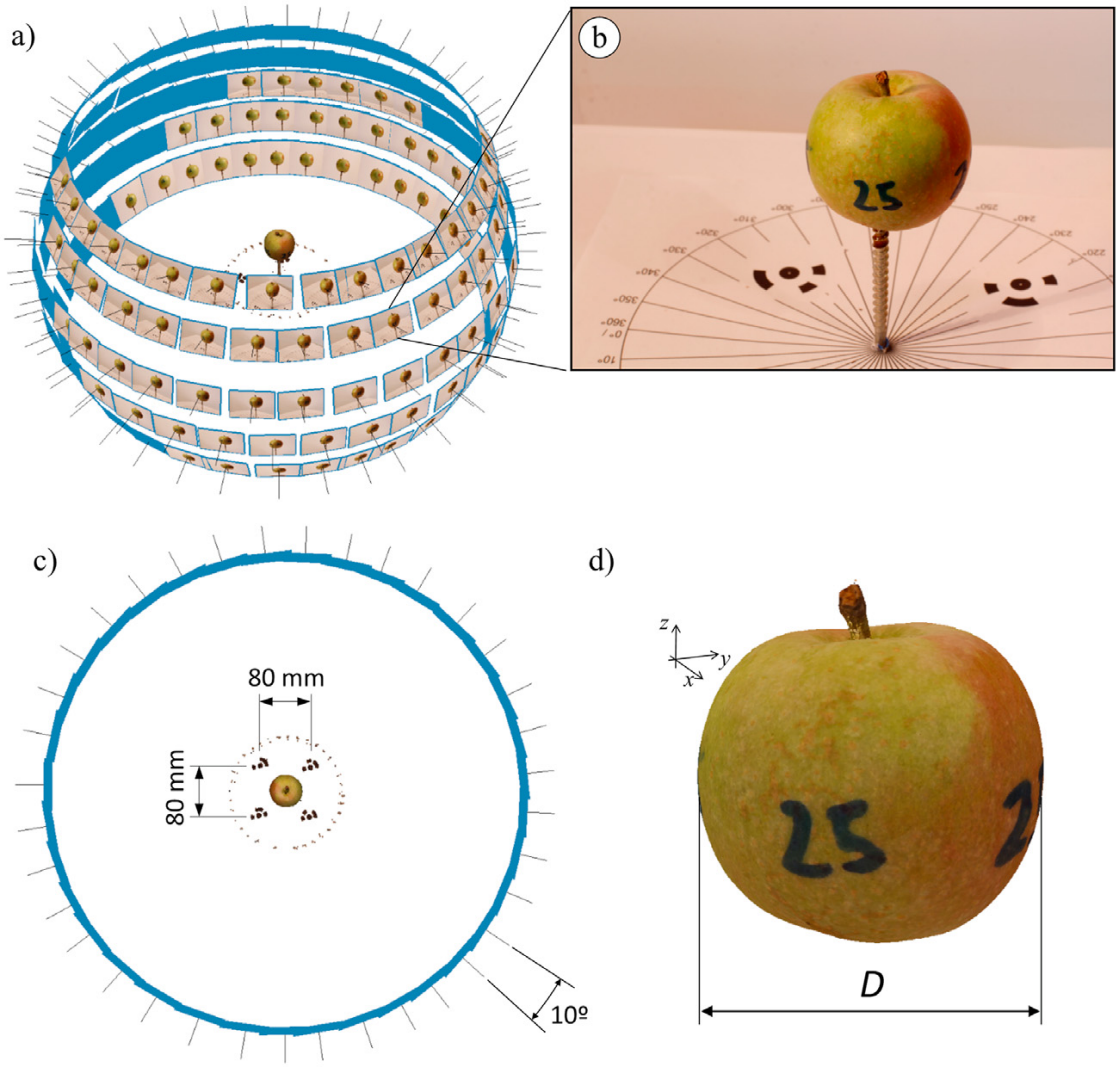
Photographic process layout followed to scan apples in laboratory conditions. (a) Isometric view showing the camera positions around the scanned apple. (b) Example of image captured during the scanning. c) Top view showing the distance between the reference markers and the angle shift between consecutive photographs. d) 3D model of an apple showing the distance measured as diameter ground truth.

The camera used to acquire the *Lab2020* set was the same as the one described for the *Orch2018* and *Orch2020* sets. However, the experimental layout was designed to obtain a high resolution 360° representation of the apples. Each apple was held in a vertical screw mounted on a turntable. Images were acquired from 5 different camera heights: −10 cm, 0 cm, 10 cm, 20 cm and 30 cm with respect to the apple centre (Fig. 5a). From each camera height, the camera was tilt so that the apple centre appears at the centre of the image and 36 images were captured around the apple (every 10° rotation) (Fig. 5b), resulting in a total of 180 images per apple: 5 height positions * 36 images/position = 180 images.

Raw images were used to generate the 3D model of trees and apples by applying multi-view structure-from-motion based on bundle adjustment [11]. To this end, Agisoft Professional Metashape software (v1.6.4, Agisoft LLC, St. Petersburg, Russia) was used with the following settings: camera alignment accuracy, “highest”; key point limit, “40000”; tie point limit, “4000”; dense cloud quality, “high”; depth filtering, “Mild”. A set of reference markers were used to scale the generated point clouds. The markers used in the laboratory scanning (*Lab2020*) were placed on the turntable 80 mm apart (Fig. 5c). The markers used in *Orch2018* and *Orch2020* were placed at the bottom part of the trees (on the ground) 850 mm apart.

**Fig. 6:**
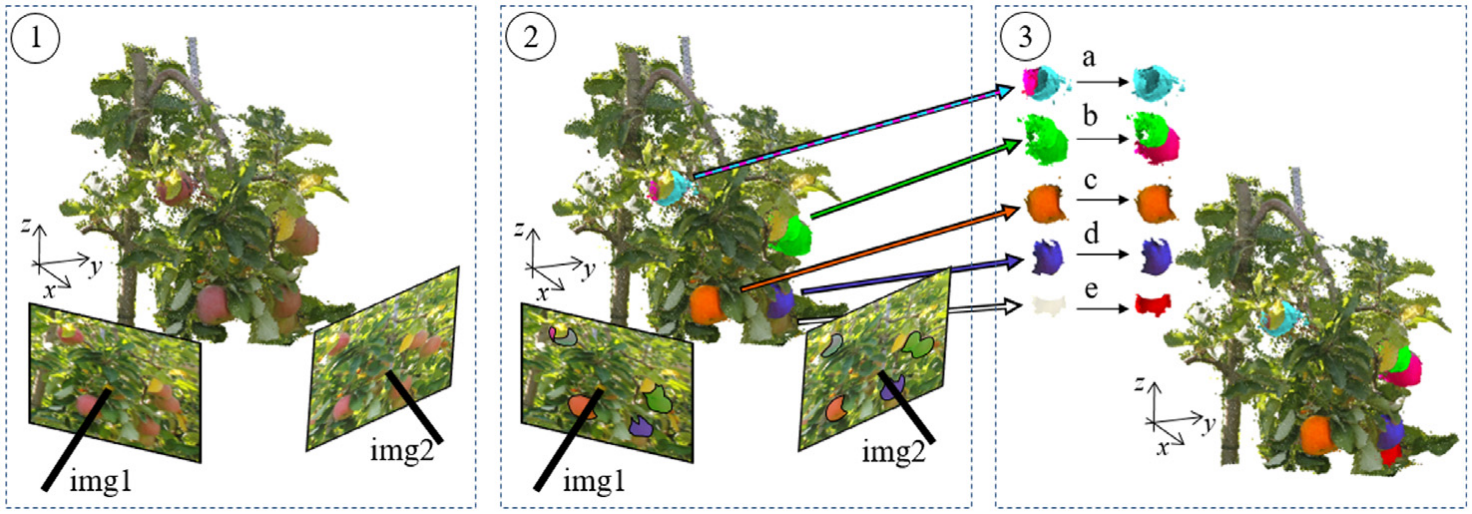
Apples segmentation procedure. (1) First, the 3D point clouds were generated. (2) Then, apples were automatically segmented in the images and the segmented pixels were projected on the point cloud. (3) Finally, the point cloud segmentation was manually corrected by merging apple multi-detections (3.a), splitting detections with more than one apple (3.b), segmenting apples not detected (3.e) and removing false positive detections.

The point clouds generated in the *Orch2018* and *Orch2020* sets were segmented in order to obtain a single point cloud per apple. To do so, a semi-automatic segmentation was carried out (Fig. 6). First, the fruit detection methodology presented in [12] was applied. This methodology consists in segmenting apples in the images and projecting detections onto the 3D point cloud (Fig. 6.2). Image instance segmentation was carried out using the Mask-RCNN [13] trained with the Fuji-SfM dataset [4]. The projection of image segmentation onto the 3D space was carried out using the pinhole camera model with intrinsic and extrinsic camera parameters calculated by Agisoft Professional Metashape software [14]. Since this method is subject to errors, the resulting segmentation was manually corrected by merging apple multi-detections (Fig. 6.3.a), splitting detections with more than one apple (Fig. 6.3.b), segmenting apples not detected (Fig. 6.3.e) and removing false positives. These manual point cloud editing processes (merge, split, segment…) were carried out using the CloudCompare software (Cloud Compare [GPL software] v2.9 Omnia).

Finally, the apple sizes were manually measured using a Vernier calliper. Since apples are not completely spherical, the measured distance was the largest horizontal diameter (Fig. 5d). Then, each measured apple was identified in the point cloud and the measurement was matched with the corresponding apple identification name (Apple ID).

## CRediT authorship contribution statement

**Jordi Gené-Mola:** Conceptualization, Methodology, Formal analysis, Investigation, Data curation, Writing – original draft, Visualization. **Ricardo Sanz-Cortiella:** Conceptualization, Investigation, Resources, Writing – review & editing. **Joan R. Rosell-Polo:** Investigation, Writing – review & editing, Project administration, Funding acquisition. **Alexandre Escolà:** Investigation, Writing – review & editing, Project administration, Funding acquisition. **Eduard Gregorio:** Investigation, Writing – review & editing, Supervision.

## Declaration of Competing Interest

The authors declare that they have no known competing financial interests or personal relationships which have or could be perceived to have influenced the work reported in this article.
